# Effects of Peroxide and Sulfur Curing Systems on Physical and Mechanical Properties of Nitrile Rubber Composites: A Comparative Study

**DOI:** 10.3390/ma17010071

**Published:** 2023-12-22

**Authors:** Hamed Peidayesh, Zuzana Nógellová, Ivan Chodák

**Affiliations:** Polymer Institute, Slovak Academy of Sciences, Dúbravská cesta 9, 845 41 Bratislava, Slovakia; zuzana.nogellova@savba.sk (Z.N.); ivan.chodak@savba.sk (I.C.)

**Keywords:** acrylonitrile butadiene rubber, composite, cross-link density, peroxide curing, sulfur curing

## Abstract

This study compares the effect of sulfur and dicumyl peroxide (DCP) vulcanizing systems on the physical and mechanical properties of rubber compounds based on acrylonitrile butadiene rubber (NBR). NBR compounds cured by different amounts of DCP and NBR vulcanizates filled with various concentrations of carbon black (CB) and a constant amount of sulfur or DCP were prepared. The vulcanizates were characterized by tensile testing, dynamic mechanical thermal analysis (DMTA), and cross-link density determination. The tensile strength and Young’s modulus were found to increase with the rising amount of DCP and CB, while elongation at break decreased. The samples vulcanized by the sulfur system and filled with CB show a substantial increase in tensile strength from 13.1 to 21.2 MPa. Higher storage modulus and glass transition temperature were observed with the increase in the amount of peroxide and filler, and consequently, the increase in cross-link density, indicating rigidity increase and lower molecular mobility. The changes in the physical and mechanical properties of the NBR vulcanizates were in correlation with the changes in solvent uptake and cross-link density.

## 1. Introduction

Rubber composites have attracted much attention for their broad application in petroleum exploitation, aerospace seals, the automotive industry, and many other fields. As industrial demands grow fast, exploring properties and testing methods of rubber compounds need to be promoted, resulting in the expansion of their applications in sealing [1,2], high-temperature resistance products [3,4,5], electroconductive materials [6,7], toughening the thermoplastics [8] and rubbers with high wear resistance [9].

It is well-known that rubbers are characterized by their ability to be reversibly deformed under external forces known as elasticity. In this regard, every rubber product undergoes a vulcanization process, which may be the most important process in the rubber industry [10,11]. In this process, a highly elastic rubber product is fabricated by forming a three-dimensional cross-linked network structure within the rubber [10,11]. The presence of active functional groups and molecular structure determines the applied curing system. Several curing strategies have been developed for the formation of 3D cross-linked networks composed of covalent and non-covalent bonds [12,13,14,15,16], including peroxides, sulfur, phenolic resins, metal oxides, and quinones. Sulfur and peroxide systems are the most frequently used curing systems for the vulcanization of rubber compounds, leading to sulfidic and carbon-carbon cross-links, respectively [17,18,19]. Peroxide vulcanization provides physical properties such as low compression test, high modulus, and resistance to thermo-oxidative aging, which corresponds to short-length and high bond energy (348 kJ/mole) of C–C bonds in comparison with C–S_X_–C bonds (273 kJ/mole) [20]. However, during the peroxide vulcanization, a number of side reactions can occur. One of these is chain scission, leading to breaking the macroradical and leaving a double bond and a radical, which results in polymer degradation and, consequently, deterioration of mechanical properties. Polymer structure plays an important role in the occurrence of scission reactions, while peroxide concentration and process temperature can also contribute to the vulcanization process [21]. Some polymers, such as polypropylene, have a structure that is more disposed to scission than cross-linking. Coagents are useful in minimizing scission reactions by stabilizing the polymer radicals. However, some polymers cannot be effectively cured by peroxides, even with coagents. Regarding the peroxide concentration, scission reaction predominates over cross-linking reaction in polypropylene at low peroxide concentration. Higher processing temperatures may also favor scission reaction [21]. On the other hand, sulfur-cured vulcanizates present high tensile and tear strength and good elastic behavior but inferior resistance to aging and low thermal stability [11]. Saturated rubbers such as ethylene–propylene rubber, silicone rubber, etc., can be vulcanized only by peroxides. In the case of unsaturated rubbers such as styrene–butadiene rubber (SBR), natural rubber (NR), acrylonitrile butadiene rubber (NBR), and butadiene rubber (BR), peroxide radical may react either by abstraction of hydrogen (allylic being much more reactive compared to alkylic) or by addition to a double bond [22,23].

NBR is a copolymer produced by emulsion polymerization of acrylonitrile and butadiene. It has been widely used in sealing products for the oil and gas industry due to its excellent oil resistance properties. However, their service life might be affected by the presence of reactive groups such as double bonds (-C=C-) and nitrile groups (-CN). In designing NBR vulcanizate for a given purpose, mutual correlations between processing, cross-linking efficiency, physical and mechanical properties, and performance should be considered. In this regard, NBR vulcanizates with excellent mechanical properties need to be optimized by selecting the proper reinforcing filler and vulcanization system. The influence of sulfur and peroxide curing systems on physical and mechanical properties of various elastomers such as SBR [24,25], NR [26,27,28,29], NBR [11,30,31], and ethylene–propylene–diene terpolymer (EPDM) [32,33] have been studied in previous works. However, only a few studies have systematically focused on the comparison of the effect of peroxide and sulfur curing systems on the physical and mechanical properties of NBR rubber [22]. Therefore, in this paper, we focused on a comparison of properties of NBR vulcanized either by classical sulfur vulcanizing system or by thermal decomposition of organic peroxide. To discuss the results on an exact basis, all properties compared for differences between the two vulcanizing systems were evaluated in relation to calculated cross-link densities determined from data of equilibrium swelling, according to the Flory–Rehner equation. The mechanical properties were investigated using dynamic mechanical thermal analysis (DMTA) and tensile testing. As far as we are aware, in the published literature, the effectiveness of the two vulcanizing systems for NBR was not compared in all details, discussing the properties in relation to cross-link density.

## 2. Materials and Methods

### 2.1. Materials

Acrylonitrile butadiene rubber (NBR, SKN 3345, Sibur International, Moscow, Russia) containing 31–35% acrylonitrile was used as a rubber matrix. Carbon black (Vulcan^®^ N–234, Cabot Corp., Lešná, Czech Republic) was used as the filler. The sulfur vulcanizing system consisted of stearic acid (Setuza, Ústí nad Labem, Czech Republic) and zinc oxide (Slovlak, Košeca, Slovakia) as activators, N-cyclohexyl-2-benzothiazole sulfenamide CBS (Duslo, Šaľa, Slovakia) as the accelerator, and sulfur (Siarkopol, Tarnobrzeg, Poland) as curing agent. Dicumyl peroxide (DCP, Merck Schuchardt, Hohenbrunn, Germany), a free-radical initiator, was used as a peroxide curing agent. Acetone for analysis grade was supplied by Mikrochem (Pezinok, Slovakia).

### 2.2. Compounding and Curing Process of Rubber Composites

The compounded mixtures with different amounts of carbon black (CB) and curing agents were prepared by two-step mixing of rubber, CB particles, and vulcanizing system for the sulfur curing system and one-step mixing for the peroxide curing system in a 50 mL laboratory mixer Plastograph PLE 331 (Brabender, Duisburg, Germany). In mixture with the peroxide system, all the components were mixed in one step for 10 min at 50 °C and 30 rpm. On the other hand, in the sulfur system, the rubber, CB, and activators (ZnO and stearic acid) were compounded in the first step for 10 min at 50 °C and 30 rpm, whereas N-cyclohexyl-2-benzothiazole sulfenamide (CBS) and sulfur were added in the second step performed for 4 min at 50 °C and 30 rpm. Further mixing was carried out for all samples using a calendering process at room temperature to increase the homogeneity using a laboratory two-roll mill (Nishimura, Tokyo, Japan). The calendering process was performed through at least ten steps consisting of sheeting, rolling, and sheeting. Two sets of NBR compounds were prepared. First, NBR compounds without any filler and with three various contents of 1, 2, and 3 phr (parts per hundred parts of rubber) peroxide curing agent, and second, NBR compounds filled with three different CB concentrations of 20, 35, and 50 phr, the concentration range is broad enough to indicate the change trends but not affecting substantially the chemistry of the vulcanization process, e.g., excessive adsorbing the components of the vulcanizing system. A constant amount of 3 phr sulfur or peroxide was used as a cross-linker. The composition and sample codes of NBR compounds are presented in Table 1. The digit after the codes indicates the concentrations of each particular component.

The curing process of NBR compounds was carried out by compression molding using a laboratory press (Fontijne TP 50, Delft, The Netherlands) at 175 °C for 20 min. The vulcanized specimens were kept in PE bags for 24 h before testing.

### 2.3. Mechanical Properties

The tensile properties of NBR vulcanizates were measured using an Instron 3365 universal testing machine (Instron, Norwood, MA, USA) at a cross-head speed of 50 mm·min^–1^ in uniaxial deformation at room temperature. In accordance with the valid technical standards, the dumbbell-shaped test specimens were used with initial dimensions of the deformed part 30.0 mm length, 4.0 mm width, and thickness of approximately 1 mm exactly measured for each testing specimen before mechanical testing. The mean values and standard deviations were calculated from seven specimens for all parameters.

### 2.4. Dynamic Mechanical Thermal Analysis (DMTA)

Dynamic–mechanical performances of the vulcanizates were investigated using a dynamic–mechanical analyzer DMA Q800 (TA Instruments, Hüllhorst, Germany). The samples (ca. 10 × 7 × 1 mm^3^) were measured at a frequency of 10 Hz and an amplitude of 20 μm in tensile mode. The temperature range of the experiment was from –80 °C to 100 °C with a heating rate of 2 °C·min^−1^.

### 2.5. Determination of Cross-Link Density

The cross-link density of NBR vulcanizates was measured by the determination of the equilibrium swelling of samples in a suitable solvent. Triplicate specimens of each NBR vulcanizate were swelled in acetone for 48 h, indicated by the equilibrium reached between 24 and 48 h at ambient temperature determined in a separate swelling measurement at various times. The specimens were taken out from the solvent and were weighed after the fast removal of acetone from the surface. The swelling degree (Q, in wt%) of the rubber composites was determined using the gravimetric method by Equation (1):

(1) $$Q\;(\mathrm{wt}\;\%)=\frac{m-m_{0}}{m_{0}}\times 100$$

where *m_0_* and m refer to the sample weight before and after swelling, respectively.

Based on the obtained equilibrium swelling measurements, the Flory–Rehner equation (Equation (2)) was applied to calculate the cross-link density, *ν*, in mol·m^–3^.

(2) $$-\ln\left(1-v_{r}\right)-v_{r}-\chi v_{r}^{2}=2\rho v_{s}vv_{r}^{\frac{1}{3}}$$

where *ρ* is the rubber density, *ν_r_* is the volume fraction of swollen rubber, *χ* is the Huggins rubber–solvent interaction parameter (for NBR–acetone *χ =* 0.3692 [11]), and *ν_s_* is the molar volume of solvent (for acetone *ν_s_* = 73.52 mL·mol^−1^). The *ν_r_* is given by Equation (3):

(3) $$v_{r}=\frac{\left(\frac{w_{d}}{\rho_{r}}\right)}{\left(\frac{w_{d}}{\rho_{r}}\right)+\left(\frac{w_{s}-w_{d}}{\rho_{s}}\right)}$$

where *w_d_* and *w_s_* are the weights of dry and swollen sample, respectively, *ρ_s_* and *ρ_r_* is the density of the solvent (for acetone *ρ_s_ =* 0.7845 g·cm^−3^), and of rubber, respectively.

## 3. Results and Discussion

### 3.1. Solvent Uptake and Cross-Link Density

The cross-link density of rubber vulcanizates is calculated using the Flory–Rehner equation according to the swelling degree of samples in the selected organic solvent. In our case, the selected solvent was acetone, and the amount of solvent uptake and calculated cross-link density of NBR vulcanizates are summarized in Table 2. It is seen that the acetone uptake is decreasing, indicating rising cross-linking density with the increase of peroxide content in vulcanizates without CB. Considering the samples with the rising content of CB, first, CB has quite a substantial effect on the cross-link density, since if comparing the sample P3 with P3-CB50, the trifold increase in cross-link density has been found and even only 20 phr of CB results in almost double value of cross-link density compared to the sample without CB (P3 vs. P3-CB20). This means that the CB surface interacts with the rubber by attaching the rubber macromolecules and forms additional physical or physico-chemical cross-links.

If we compare the data on the cross-link density of sulfur-vulcanized with the peroxide-vulcanized NBR, the latter system initiates a formation of significantly higher cross-link density. The addition of 20 phr of CB into the vulcanizate cross-linked by sulfur leads to a formation of cross-link density that is only marginally higher compared to sample P3 without CB cross-linked with peroxide. A comparison of samples vulcanized by sulfur and peroxides with the same CB content reveals that the cross-link density formed by peroxide curing is more than 50% higher than if sulfur vulcanization is performed. Finally, the comparison of cross-link density as well as all corresponding parameters (e.g., T_g_) shows that for NBR without CB, cross-linked by 1 phr of peroxide, the cross-link density is comparable with the sample cross-linked by 3 phr of sulfur.

The following clarification is offered to explain this difference. First, the same weight portion of sulfur and peroxide were added. The molecular weight of DCP is 270.37 g·mol^−1,^ and one molecule initiates two macroradicals, consequently forming one cross-link. The mass weight of sulfur is 32 g·mol^−1^. However, sulfur rarely forms monosulfidic bonds during vulcanization. To initiate the same number of cross-links as DCP, the polysulfidic cross-links should be formed on average by eight sulfur atoms, which looks like a realistic estimate [11]. Moreover, the process of sulfur vulcanization does not assume any chain mechanism to increase the number of cross-links. On the other hand, peroxide-initiated curing in the case of NBR may lead to a chain mechanism of cross-link formation, which occurs by the addition of one macroradical to double bond of rubber chain, forming the first junction and leaving one free radical, keeping the chain reaction to continue further until termination step occurs either by abstraction of hydrogen from hydrocarbon chain or by recombination of two adjacent macroradicals.

It has to be mentioned that besides chemically formed cross-links by the formation of either sulfuric bonds or covalent C–C bonds, the cross-link density may also either increase due to the formation of entanglements without chemical bonds formation or decrease by a kind of side reaction resulting in bond scission. A decrease in expected cross-link density may also result from a formation of intramolecular bonds when each intramolecular bond consumes one molecule of peroxide or the corresponding number of sulfur atoms, but such bonds do not contribute to changes of physical properties (so-called “elastically inactive bonds”) since the bond is connecting two carbons of the same polymeric chain, so instead of connecting two polymeric chains resulting in an increase in molecular weight, a loop on the same chain appears which does not contribute to the increase of any strength parameter, such as tensile strength, elongation at break, Young’s modulus or moduli M100 or M300.

Considering the cross-link density, the presence of CB must also be considered. The rather strong reinforcing effect of the CB consists mainly of a strong interaction between the CB surface and rubber macromolecules, causing the polymer chains to attach to the CB surface. If two different macromolecules are attached to the same CB particle, the latter forms a kind of physical or physico-chemical cross-link junction. A rather frequent occurrence of such a mechanism is proven by the formation of so-called “bound rubber”, which can be determined according to the formation of an insoluble portion of non-vulcanized CB-filled rubber left after equilibrium extraction of filled rubber by a good solvent that dissolves unfilled virgin rubber completely. The amount of insoluble remnant after such extraction depends only on the CB content in the rubber. Of course, the content of bound rubber determined by different solvents may vary according to a particular solvent since the formed physical bonds are created by a number of different reactive sites between both unvulcanized rubber and CB surface; therefore, the destruction of physical bonds of different strength between CB and rubber depends on the ability of a particular solvent [34,35].

### 3.2. Mechanical Properties

The mechanical properties of NBR vulcanizates are summarized in Table 3. It is seen that the tensile strength and Young’s modulus of the samples without CB (P1–P3) cured by peroxide increase slightly with the rising peroxide content while elongation at break decreases. The same trend can be seen for the NBR vulcanizates filled with various amounts of CB, exhibiting a more pronounced increase in tensile strength and Young’s modulus. On the other hand, the samples vulcanized by the sulfur system and filled with CB show a substantial enhancement in tensile strength from 13.1 to 21.2 MPa, while Young’s modulus increased from 5.8 to 12.1 MPa. Moreover, as seen in Table 3, the elongation at break values is much higher compared to the vulcanizates cured by peroxide.

It is well-known that the vulcanization system and type of rubber determine not only the level of cross-link density but also the effect of each particular cross-link on the physical, especially mechanical properties of the vulcanizate. Thus, shorter and stronger C–C bonds formed during peroxide-initiated vulcanization create stronger but less mobile three-dimensional cross-linked structures compared to longer and more flexible cross-links formed by polysulfidic cross-bonds connecting the rubber macromolecules. The mechanical properties presented in Table 3 are fully in line with this explanation. Basically, increasing the level of sulfur or peroxide leads to an increase in cross-link density since more C–C bonds in the peroxide curing system and more C–S_X_–C linkages in the sulfur system are formed [22]. As seen in Table 3, the NBR vulcanizates cured by the sulfur system show higher tensile stress compared to the samples cured by peroxide. This is mainly attributed to the more flexible cross-link network of sulfur-vulcanized NBR enabling higher elongation at break, resulting in more extensive orientation of rubber macromolecules during unidirectional deformation, compared to much stiffer peroxide-vulcanized rubber, which exhibits higher Young’s moduli but breaks at lower load when the tensile stress value is smaller than the one for sulfur-vulcanized elastomer.

**Figure 1:**
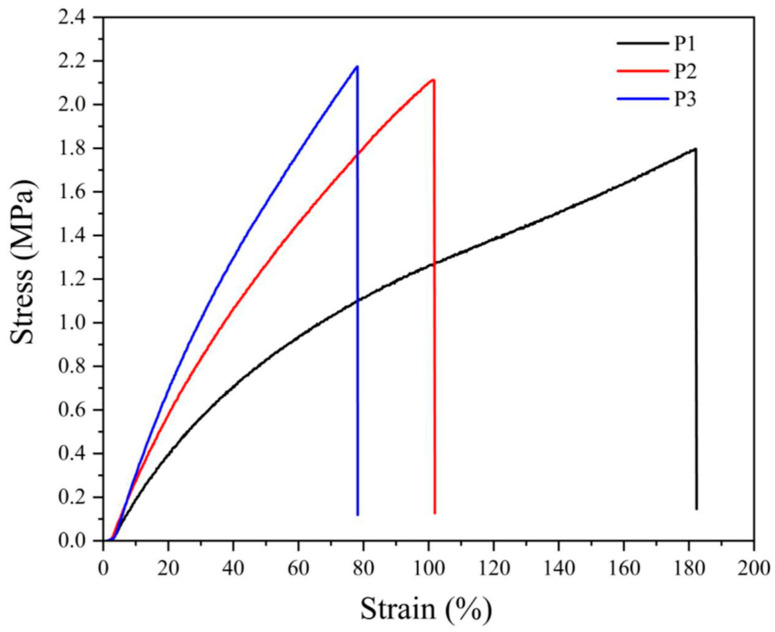
Stress–strain curves for neat NBR vulcanizates cured by various amounts of dicumyl peroxide (P), the digit after the code P indicates its concentration in phr.

**Figure 2:**
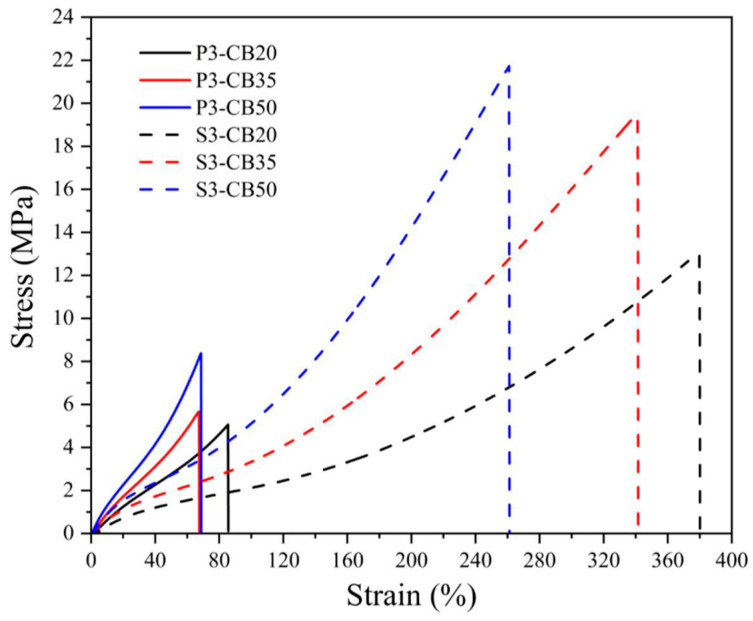
Stress–strain curves for NBR vulcanizates filled with different carbon black (CB) concentrations and cured by 3 phr of dicumyl peroxide (P) or sulfur (S). The digit after the codes indicates the concentration in phr.

The stress–strain behavior of NBR vulcanizates presented in Figure 1 and Figure 2 confirm the previous considerations, especially concerning a much steeper increase in stress of peroxide-vulcanized CB-filled materials that break at substantially lower loads.

**Figure 3:**
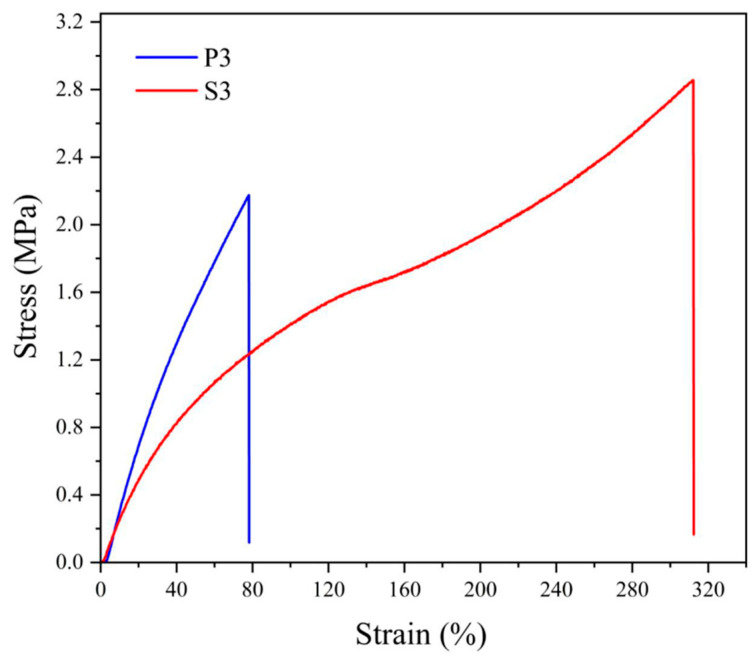
Stress–strain curves for neat NBR vulcanized by 3 phr of dicumyl peroxide (P) or vulcanizing system with 3 phr of sulfur (S), the digit after the codes indicates the concentration in phr.

The differences in the behavior of NBR cross-linked by sulfur vulcanizing system or by peroxide can be explained in the simplest way by comparing the stress–strain curves for both materials, shown in Figure 3.

Typical differences consist of a faster increase in strength at the beginning of deformation for peroxide-vulcanized NBR and substantially higher deformation of the specimen for sulfur-vulcanized rubber. Since both materials differ only in the vulcanizing system, the structure of the cross-bonds is decisive considering the effect of cross-linking. In the case of peroxide vulcanization, the cross-links consist of covalent bonds C–C connecting two macroradicals of rubber, while during sulfur vulcanization, the cross-link is formed by a sulfur atom (usually only if a special vulcanizing system is used) or by short chain formed by several (2 to 4 or even more) sulfur atoms. The difference in behavior consists in the fact that the C–C bond is shorter and more rigid compared to sulfuric multiatom cross-link. Therefore, peroxide-vulcanized rubber has higher rigidity but smaller deformation, while sulfur-vulcanized rubber possesses the opposite behavior.

Similar behavior is typical also for the rubber reinforced by fillers, e.g., carbon blacks. just in that case, some additional effects are observed, such as an increase in the cross-link density due to the formation of physical cross-links by attaching the rubber chains on the filler surface, a higher portion of entanglement (both increasing cross-link density), or due to the adsorption of sulfur or deactivation of oxyradicals on the CB surface or in pores leading to a decrease of cross-link density.

### 3.3. Dynamic Mechanical Thermal Analysis (DMTA)

**Figure 4:**
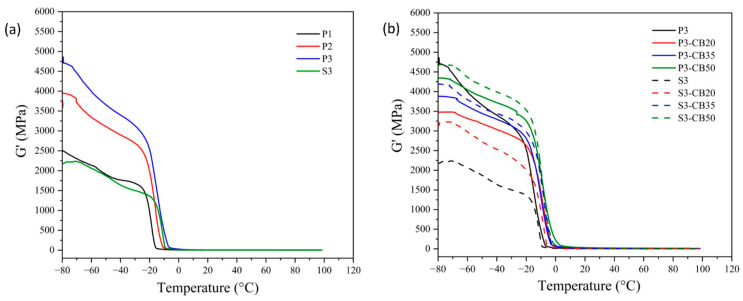
Temperature dependences of the storage modulus for (**a**) neat NBR vulcanizates cured by dicumyl peroxide (P) or sulfur vulcanizing system (S) and (**b**) for NBR vulcanizates filled with different carbon black (CB) concentrations and cured by 3 phr of dicumyl peroxide or sulfur. The digit after these codes indicates the concentration in phr.

**Figure 5:**
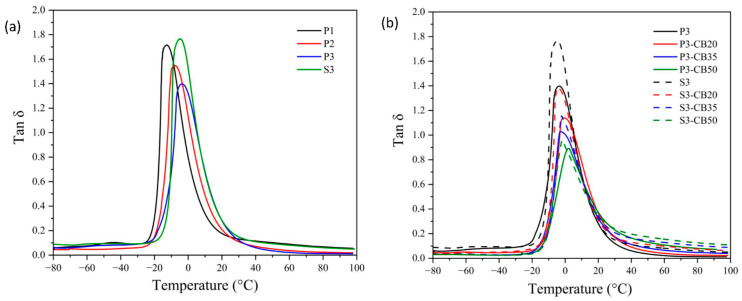
Temperature dependences of Tan δ for (**a**) neat NBR vulcanizates cured by dicumyl peroxide (P) or sulfur vulcanizing system (S) and (**b**) for NBR vulcanizates filled with different carbon black (CB) concentrations and cured by 3 phr of dicumyl peroxide or sulfur. The digit after these codes indicates the concentration in phr.

DMTA analysis is the most frequently used method for the evaluation of the viscoelastic behavior of amorphous polymers and elastomers. It enables the quantitative estimate of the motions of the macromolecule segments as well as the internal changes in the structure. The data of storage modulus and tan δ of the NBR vulcanizates as a function of temperature are shown in Figure 4 and Figure 5, respectively. In Table 2, the dependence of the glass transition temperature (T_g_) on cross-link density is presented for the investigated vulcanizates. As expected, it demonstrates that a growing amount of the peroxide results in rising cross-link density, leading to the increase in the storage modulus and consequently to a shift of T_g_ values of the vulcanizates to higher temperatures, indicating a rigidity increase.

Considering the effect of CB content, rising concentration consists of an increase of the storage modulus attributed to the reinforcement effect of the presence of CB particles. As mentioned before, CB particles may act as a physical cross-linker and, consequently, support the increase of the glass transition temperature of vulcanizate [36,37]. However, the opposite effect due to the agglomeration of CB particles should be considered. In any case, the effect of the increase in CB content on T_g_ is significantly lower compared to the increase in vulcanizing agent (peroxide) concentration, although the changes in cross-linking density are higher.

**Figure 6:**
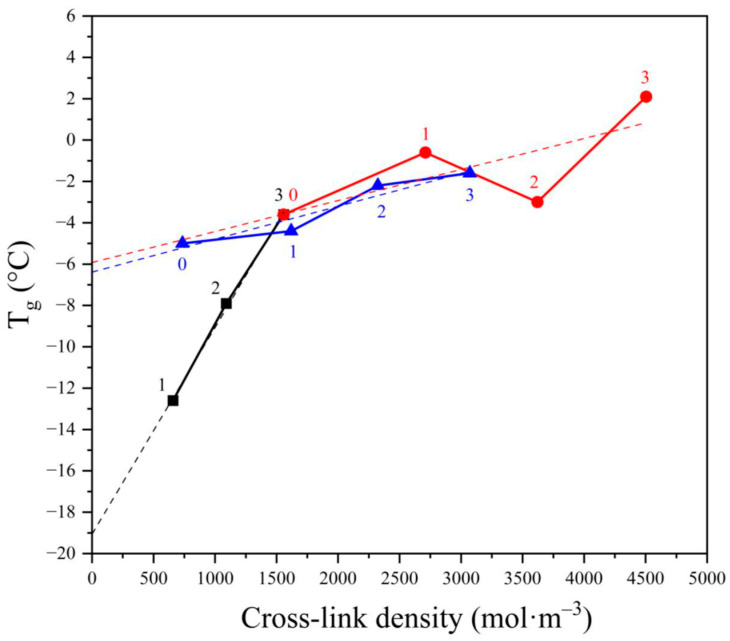
The dependence of glass transition temperature (T_g_) on the cross-link density for NBR vulcanized by various amounts of dicumyl peroxide (black, numbers indicate the peroxide concentration in phr) and vulcanized by 3 phr of either peroxide (red) or sulfuric system (blue) filled with varying amounts of carbon blacks (0—0 phr, 1—20 phr, 2—35 phr, and 3—50 phr).

However, the considerations presented above do not explain unambiguously the effect of cross-links with the increase of the cross-linking agent concentration and the quantitative contribution of CB presence. This discussion can be extended according to the data in Figure 6, where the dependence of glass transition temperature T_g_ on the cross-link density is shown.

First, it can be said that the dependence of T_g_ on the cross-link density is linear for the peroxide vulcanized NBR without CB. This could be expected since the cross-link density should depend linearly on the peroxide concentration if the decomposition of peroxide is of a monomolecular nature, which is the case for DCP in apolar polymers [38].

The linear dependence is also seen in Figure 6 (black line). Moreover, the extrapolation of the line to zero peroxide concentration indicates the T_g_ for nonvulcanized NBR around −19 °C, which seems reasonable since the published T_g_ is −25 °C. The difference between published T_g_ and our experimental T_g_ may be ascribed to a certain extent to the difference in the composition since the published value counts for virgin NBR while extrapolation of our results to zero peroxide concentration was performed according to NBR after processing in the internal mixer where a small decrease in the molecular weight could occur leading to a small increase in T_g_.

It is clearly seen that the slope of the dependence for the NBR vulcanized by peroxide is substantially higher (0.01) compared to both NBRs with varying amounts of CB vulcanized either by sulfur or by peroxide, which were found to be 0.0019 and 0.0015, respectively. The apparent coincidence of the slopes of the two NBRs vulcanized by different vulcanizing agents may help us to discuss the obvious discrepancy in the “linearity” of the CB containing NBR vulcanized by peroxide (red line). While the linearity of the dependence of sulfur vulcanized NBR–CB composite may be accepted, the scatter of the dependence for peroxide vulcanized NBR is behind any reasonable limit. However, the computer-calculated slope for both CB-containing vulcanizates is surprisingly similar, and it is the same if the sample containing 35 phr of CB has been eliminated. Considering this, we dare to discuss all measured data concerning the effect of the concentration of peroxide and the content of CB on the dependence of cross-link density on T_g_.

Thus, Figure 6 supports the outcomes in previous paragraphs, concluding that T_g_ values depend on the cross-linking density of the vulcanizates, but considering not only chemical cross-links created directly during the vulcanization but also physical cross-links, precursors of which are formed during the processing procedure which may affect the number of entanglements acting as physical elastically active junctions. Considering the slopes of the two lines representing the NBR filled with CB and cross-linked either with peroxide or with sulfur, the slopes for both lines are almost the same, indicating that the contribution of CB to cross-link density does not depend on the vulcanizing system. Thus, the main difference in the two vulcanizing systems consists in the efficiency of vulcanization, where the decomposition of one molecule of DCP forms more than one cross-link. This can be explained by suggesting the following mechanisms of the two cross-linking processes. The reaction of NBR double bond with sulfur results in the formation of one sulfuric short chain grafted on the rubber chain, where the other end of the sulfuric chain reacts with another rubber macromolecule, forming one cross-link. In the case of peroxidic vulcanization, one cumyloxy radical may abstract hydrogen from the NBR, forming a macroradical, which may either react with another macroradical by recombination reaction forming one cross-link, or decay by addition reaction to another double bond forming, besides cross-link, another macroradical. This may either terminate by recombination or start a chain reaction by addition to another double bond, forming one cross-link and another macroradical able to terminate by recombination or add to another double bond so that one primary radical created by decomposition of one molecule of DCP may initiate a formation of several cross-links. In such a way, a selection of a particular vulcanizing agent may lead to different cross-link densities and, consequently, to different ultimate properties of the vulcanizates.

## 4. Conclusions

The comparison of the physical properties of NBR-based vulcanizates cured by organic peroxide and sulfur vulcanizing system revealed that both tensile strength and elongation at break are significantly higher when NBR is vulcanized by sulfur and accelerator system when compared with curing by dicumyl peroxide while the stiffness of the latter is higher.

The increase of the peroxide content results in an increase of cross-linking density and consequently also in the rise of glass transition temperature (T_g_). The addition of carbon black acting as a reinforcing filler leads to an increase in tensile strength and to a marginal decrease of elongation at break, corresponding to an increase in cross-link density. The cross-link density in NBR vulcanized by peroxide is significantly higher compared to NBR cured by the sulfuric system. However, the effect of T_g_ increase is lower for CB changes than for vulcanizing agent content.

The possible applications of NBR rubber are important in the case of high resistance toward organic solvents. Flexible sealing rings are often applied to keep organic liquids (e.g., waxes) to transfer the stress, heat, or electricity while needing to compensate for volume changes due to heating–cooling cycles of the environment. The selection of an appropriate vulcanization system may be crucial to get good performance.

## Figures and Tables

**Table 1:** Composition of NBR compounds. The code letters S, P, and CB represent sulfur, dicumyl peroxide, and carbon black, respectively, the digit after the code letter indicates the amount of each component in phr. CBS and DCP represent N-cyclohexyl-2-benzothiazole sulfenamide and dicumyl peroxide, respectively.

Component	P1	P2	P3	S3	P3-CB20	P3-CB35	P3-CB50	S3-CB20	S3-CB35	S3-CB50
Rubber	100	100	100	100	100	100	100	100	100	100
CB	0	0	0	0	20	35	50	20	35	50
ZnO	0	0	0	3	0	0	0	3	3	3
Stearic acid	0	0	0	1	0	0	0	1	1	1
CBS	0	0	0	1	0	0	0	1	1	1
Sulfur	0	0	0	3	0	0	0	3	3	3
DCP	1	2	3	0	3	3	3	0	0	0

**Table 2:** The equilibrium solvent uptake, calculated cross-link density, and glass transition temperature (T_g_) of NBR composites vulcanized by dicumyl peroxide (marked P) or by sulfur-accelerator system (marked S) without filler or containing carbon blacks (CB). The digits after each character indicate the content in the phr of each particular mixture component.

Sample Code	Solvent Uptake (%)	Cross-Link Density ν (mol·m^–3^)	Glass Transition T_g_ (°C)
P1	181 ± 14	658 ± 20	−12.6
P2	135 ± 3	1091 ± 16	−7.9
P3	109 ± 7	1557 ± 13	−3.6
P3-CB20	77 ± 2	2710 ± 34	−0.6
P3-CB35	64 ± 3	3621 ± 19	−3.0
P3-CB50	55 ± 2	4504 ± 7	2.1
S3	170 ± 4	735 ± 15	−5.0
S3-CB20	107 ± 8	1619 ± 21	−4.4
S3-CB35	85 ± 3	2323 ± 14	−2.2
S3-CB50	71 ± 2	3071 ± 24	−1.6

**Table 3:** Mechanical properties, including tensile strength, elongation at break, Young’s modulus, and moduli M100, M200, and M300 for the NBR vulcanizates, the code letters S, P, and CB represent sulfur, dicumyl peroxide, and carbon black, respectively and the digit after the codes show their concentration in phr.

Sample Code	Tensile Strength (MPa)	Elongation at Break (%)	Young’s Modulus (MPa)	M 100 (MPa)	M 200 (MPa)	M 300 (MPa)
P1	1.8 ± 0.1	179 ± 14	2.3 ± 0.1	1.3 ± 0.1	n/a *	n/a
P2	2.0 ± 0.1	102 ± 7	3.4 ± 0.1	2.0 ± 0.1	n/a	n/a
P3	2.2 ± 0.2	78 ± 12	4.4 ± 0.1	n/a	n/a	n/a
P3-CB20	5.0 ± 1.2	83 ± 16	8.3 ± 0.8	n/a	n/a	n/a
P3-CB35	5.9 ± 1.8	68 ± 16	12.3 ± 1.6	n/a	n/a	n/a
P3-CB50	8.0 ± 1.3	65 ± 7	17.3 ± 2.0	n/a	n/a	n/a
S3	2.8 ± 0.1	313 ± 9	2.4 ± 0.1	1.4 ± 0.0	1.9 ± 0.0	2.7 ± 0.1
S3-CB20	13.1 ± 2.3	377 ± 44	5.8 ± 0.4	2.2 ± 0.1	4.6 ± 0.1	8.8 ± 0.2
S3-CB35	19.3 ± 2.6	341 ± 27	8.8 ± 0.3	3.2 ± 0.2	8.1 ± 0.4	15.7 ± 0.7
S3-CB50	21.2 ± 5.2	255 ± 41	12.1 ± 1.2	5.3 ± 0.2	14.5 ± 0.4	n/a

* Not applicable.

## Data Availability

The data presented in this study cannot be shared at this time as they form a part of ongoing research.

## References

[1] Cui T., Chao Y., Van Zee J. (2012). Stress relaxation behavior of EPDM seals in polymer electrolyte membrane fuel cell environment. Int. J. Hydrog. Energy.

[2] Dong Y., Ke Y., Zheng Z., Yang H., Yao X. (2017). Effect of stress relaxation on sealing performance of the fabric rubber seal. Compos. Sci. Technol..

[3] Han R., Wang Z., Zhang Y., Niu K. (2019). Thermal stability of CeO_2_/graphene/phenyl silicone rubber composites. Polym. Test..

[4] Yang H., Yao X.-F., Ke Y.-C., Ma Y.-j., Liu Y.-H. (2016). Constitutive behaviors and mechanical characterizations of fabric reinforced rubber composites. Compos. Struct..

[5] Han R., Wu Y., Quan X., Niu K. (2020). Effects of crosslinking densities on mechanical properties of nitrile rubber composites in thermal oxidative aging environment. J. Appl. Polym. Sci..

[6] Peidayesh H., Špitalský Z., Chodák I. (2022). Electrical Conductivity of Rubber Composites with Varying Crosslink Density under Cyclic Deformation. Polymers.

[7] Evgin T., Mičušík M., Machata P., Peidayesh H., Preťo J., Omastová M. (2022). Morphological, Mechanical and Gas Penetration Properties of Elastomer Composites with Hybrid Fillers. Polymers.

[8] Lendvai L. (2023). Mechanical and morphological properties of PP/XNBR blends produced with rubber latex. J. Polym. Res..

[9] Li Y., Wang Q., Wang T., Pan G. (2012). Preparation and tribological properties of graphene oxide/nitrile rubber nanocomposites. J. Mater. Sci..

[10] Kruželák J., Sýkora R., Hudec I. (2016). Sulphur and peroxide vulcanisation of rubber compounds–overview. Chem. Pap..

[11] Kruželák J., Chodák I., Mošková D.J., Dosoudil R., Hudec I. (2018). Cross-linking and properties of rubber magnetic composites cured with different curing systems. Polym. Adv. Technol..

[12] Mora-Barrantes I., Malmierca M., Valentin J., Rodriguez A., Ibarra L. (2012). Effect of covalent cross-links on the network structure of thermo-reversible ionic elastomers. Soft Matter.

[13] Posadas P., Malmierca M., González-Jiménez A., Ibarra L., Rodríguez A., Valentin J., Nagaoka T., Yajima H., Toki S., Che J. (2016). ESR investigation of NR and IR rubber vulcanized with different cross-linking agents. Express Polym. Lett..

[14] Shangguan Y., Yang J., Zheng Q. (2017). Rheology of nitrile rubber with hybrid crosslinked network composed of covalent bonding and hydrogen bonding. RSC Adv..

[15] Fleischmann D.D., Ayalur-Karunakaran S., Arbeiter F., Schaller R., Holzner A., Kern W., Schlögl S. (2018). Influence of crosslinker and water on mechanical properties of carboxylated nitrile butadiene rubber (XNBR). Polym. Test..

[16] Utrera-Barrios S., Araujo-Morera J., de Los Reyes L.P., Manzanares R.V., Verdejo R., López-Manchado M.Á., Santana M.H. (2020). An effective and sustainable approach for achieving self-healing in nitrile rubber. Eur. Polym. J..

[17] Aprem A.S., Joseph K., Mathew T., Altstaedt V., Thomas S. (2003). Studies on accelerated sulphur vulcanization of natural rubber using 1-phenyl-2, 4-dithiobiuret/tertiary butyl benzothiazole sulphenamide. Eur. Polym. J..

[18] Heideman G., Noordermeer J.W., Datta R.N., van Baarle B. (2005). Effect of zinc complexes as activator for sulfur vulcanization in various rubbers. Rubber Chem. Technol..

[19] Tao Z., Viriyabanthorn N., Ghumman B., Barry C., Mead J. (2005). Heat resistant elastomers. Rubber Chem. Technol..

[20] Seghar S., Asaro L., Rolland-Monnet M., Hocine N.A. (2019). Thermo-mechanical devulcanization and recycling of rubber industry waste. Resour. Conserv. Recycl..

[21] Dluzneski P.R. (2001). Peroxide vulcanization of elastomers. Rubber Chem. Technol..

[22] El-Nemr K.F. (2011). Effect of different curing systems on the mechanical and physico-chemical properties of acrylonitrile butadiene rubber vulcanizates. Mater. Des..

[23] Valentín J.L., Rodríguez A., Marcos-Fernández A., González L. (2005). Dicumyl peroxide cross-linking of nitrile rubbers with different content in acrylonitrile. J. Appl. Polym. Sci..

[24] Ahmed S., Basfar A., Aziz M.A. (2000). Comparison of thermal stability of sulfur, peroxide and radiation cured NBR and SBR vulcanizates. Polym. Degrad. Stab..

[25] Rodak A., Susik A., Kowalkowska-Zedler D., Zedler Ł., Formela K. (2023). Cross-Linking, Morphology, and Physico-Mechanical Properties of GTR/SBS Blends: Dicumyl Peroxide vs. Sulfur System. Materials.

[26] Basfar A., Abdel-Aziz M., Mofti S. (2002). Influence of different curing systems on the physico-mechanical properties and stability of SBR and NR rubbers. Radiat. Phys. Chem..

[27] Ikeda Y., Yasuda Y., Hijikata K., Tosaka M., Kohjiya S. (2008). Comparative study on strain-induced crystallization behavior of peroxide cross-linked and sulfur cross-linked natural rubber. Macromolecules.

[28] Kruželák J., Sýkora R., Dosoudil R., Hudec I. (2017). Relationship between the cross-link structure and properties of peroxide and sulfur-cured magnetic composites based on NR and NBR. J. Elastomers Plast..

[29] Vázquez-Martínez Y., Ramírez-Herrera C.A., Mondragón M., Elías-Zúñiga A., Elizalde L.E. (2022). Effect of Single-Walled Carbon Nanotubes on the Cross-Linking Process in Natural Rubber Vulcanization. Polymers.

[30] Cong C., Liu Q., Li J., Meng X., Zhou Q. (2019). The effect of peroxide crosslinking on the synergistic crosslink of double bond and nitrile group of nitrile rubber in H2S environment. Polym. Test..

[31] Zedler Ł., Colom X., Cañavate J., Formela K. (2021). GTR/NBR/silica composites performance properties as a function of curing system: Sulfur versus peroxides. Materials.

[32] Zou H., Qiu G., Liu G., Soddemann M., Xu J. (2020). A Novel Approach to Investigating Effect of Sulfur as a Coagent on the Quasi-Static and Cyclic–Dynamic Fatigue Properties of Peroxide Cured EPDM. Polym. Eng. Sci..

[33] Ketikis P., Ketikis I., Tarantili P.A. (2023). The effect of cross-linking system and reinforcement on the cross-linking reaction of peroxide vulcanized ethylene-propylene-diene terpolymer (EPDM) matrix. J. Macromol. Sci. A.

[34] Choi S.S. (2004). Effect of bound rubber on characteristics of highly filled styrene–butadiene rubber compounds with different types of carbon black. J. Appl. Polym. Sci..

[35] Gabriel D., Karbach A., Drechsler D., Gutmann J., Graf K., Kheirandish S. (2016). Bound rubber morphology and loss tangent properties of carbon-black-filled rubber compounds. Colloid Polym. Sci..

[36] Peidayesh H., Mosnáčková K., Špitalský Z., Heydari A., Šišková A.O., Chodák I. (2021). Thermoplastic starch–based composite reinforced by conductive filler networks: Physical properties and electrical conductivity changes during cyclic deformation. Polymers.

[37] Peidayesh H., Ahmadi Z., Khonakdar H.A., Abdouss M., Chodák I. (2020). Fabrication and properties of thermoplastic starch/montmorillonite composite using dialdehyde starch as a crosslinker. Polym. Int..

[38] Bermejo J.S., Ugarte C.M. (2009). Influence of cross-linking density on the glass transition and structure of chemically cross-linked PVA: A molecular dynamics study. Macromol. Theory Simul..

